# La maladie de Fox Fordyce péri sternale: un cas rare et atypique

**DOI:** 10.11604/pamj.2019.33.1.17077

**Published:** 2019-05-02

**Authors:** Younes Barbach, Fatima Zahra Mernissi

**Affiliations:** 1 Service de Dermatologie et Vénérologie, Hôpital Universitaire Hassan II, Fès, Maroc

**Keywords:** Fox Fordyce, peristernal, apocrine anhidrosis, rare, atypical, Fox Fordyce, péri sternale, anhidrose apocrine, rare, atypique

## Abstract

Syringomas are benign tumors of cutaneous appendages of eccrine or apocrine origin affecting approximately 1% of the population. They mainly occur in women and they commonly manifest as soft skin-colored or slightly yellowish papules on the lower eyelid and the upper part of the cheeks. More rarely, syringomas can even occur on the neck, the armpits, the breasts, the lower portion of the abdomen, the thighs and the groin. Clinically, they can be distinguished from xanthelasmas, warts or cancers because they are monomorphic and have a regular distribution. In doubtful cases, the diagnosis is confirmed by biopsy. Syringomas can be easily detected on histological examination due to the presence of comma-shaped sweat ducts in the dermis. Even though syringomas are benign tumors, their appearance can be embarrassing to the patients. Therapeutic options, mainly supported by small case series and case reports, include surgical excision, electrodessication, curettage, chemical exfoliation, cryosurgery and laser treatment. However, as these tumors lie deep in the dermis, all treatments are associated with a substantial risk of recurrence and can cause scars and skin pigment changes. We here report the case of a 40-year old woman with no previous history, presenting with skin-colored periorbital papules whose histological examination showed syringomas.

## Image en médecine

La maladie de Fox-Fordyce (FF), également appelée milaria apocrine, est une maladie chronique rare caractérisée par des papules folliculaires prurigineuses localisées dans des zones glandulaires apocrines. La physiopathologie consiste en l'obstruction du canal de la glande apocrine par l'insertion d'un bouchon de kératine dans la paroi des poils, cela provoque une rétention de sécrétion avec rupture conséquente de la structure glandulaire et une inflammation secondaire du derme. L'extravasation du contenu glandulaire peut être la cause du prurit. Cliniquement, les lésions sont des papules uniformes, fermes, folliculaires, qui peuvent toucher les aisselles, les zones anogénitales et péri-aréolaires, les lèvres, l'ombilic, le périnée et plus rarement le sternum comme dans notre cas. Le diagnostic de la maladie FF est basé sur des caractéristiques cliniques caractéristiques, ainsi que des caractéristiques histopathologiques non spécifiques comprenant la dilatation de l'infundibulum folliculaire avec hyperkératose, acanthose et spongiose de l'épithélium infundibulaire et l'infiltration périfolliculaire des lymphocytes et des histiocytes, qui conduisent à la perte de cheveux. Divers traitements pour la maladie FF ont été suggérés avec une amélioration limitée, y compris l'administration de contraceptifs oraux, les corticostéroïdes topiques, intralésionnels ou systémiques, les rétinoïdes topiques et oraux, la clindamycine topique comme prescrite dans notre cas, le pimécrolimus, la photothérapie, les traitements chirurgicaux comme l'électrocoagulation et le curetage avec liposuccion. Nous rapportons le cas d’un patient âgé de 34 ans, sans antécédent pathologique notable, consultait pour la prise en charge de lésions prurigineuses du tronc évoluant depuis 5 ans. L’examen dermatologique avait objectivé la présence de multiples papules couleur chair faisant environ 0,5cm de grand axe siégeant au niveau de la région péri sternale prurigineuses et indolores. La dermoscopie montrait la présence de papules folliculaires, des poils terminaux traumatisés et des points noirs. Le patient avait bénéficié d’une biopsie cutanée revenue en faveur d’une maladie de Fox Fordyce, puis mis sous clindamycine topique.

**Figure 1:**
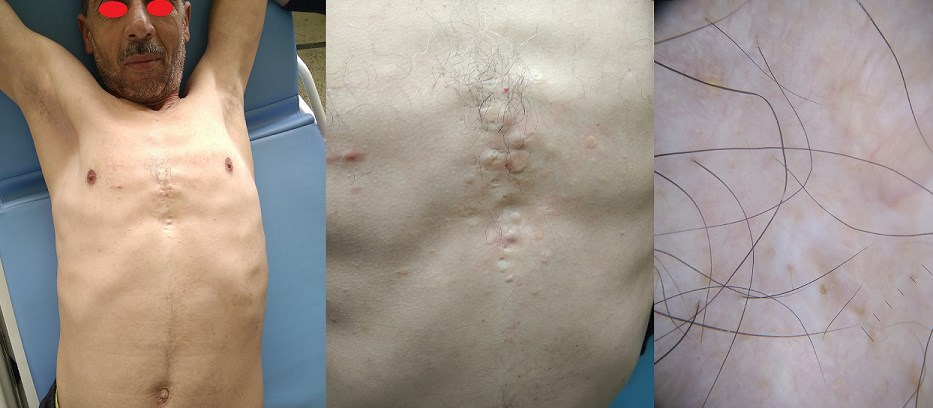
Multiples papules couleur chair au niveau de la région péri sternale

